# Resource-Efficient Cotton Network: A Lightweight Deep Learning Framework for Cotton Disease and Pest Classification

**DOI:** 10.3390/plants14132082

**Published:** 2025-07-07

**Authors:** Zhengle Wang, Heng-Wei Zhang, Ying-Qiang Dai, Kangning Cui, Haihua Wang, Peng W. Chee, Rui-Feng Wang

**Affiliations:** 1 College of Information and Electrical Engineering, China Agricultural University, 17 Qinghua East Road, Haidian, Beijing 100083, China; wangzhengle@cau.edu.cn; 2 College of Engineering, China Agricultural University, 17 Qinghua East Road, Haidian, Beijing 100083, China; hwzhang@cau.edu.cn; 3 School of Information Engineering, Inner Mongolia University of Technology, No.1 Kejian Road, Jinchuan New District, Hohhot 010080, Inner Mongolia, China; 202310201045@imut.edu.cn; 4 Department of Mathematics, City University of Hong Kong, 83 Tat Chee Ave., Kowloon, Hong Kong; kangnicui2-c@my.cityu.edu.hk; 5 Department of Crop and Soil Sciences and Institute of Plant Breeding, Genetics, and Genomics, University of Georgia, Tifton, GA 31793, USA

**Keywords:** cotton, disease and pest diagnosis, images classification, lightweight model, MobileViT

## Abstract

Cotton is the most widely cultivated natural fiber crop worldwide, yet it is highly susceptible to various diseases and pests that significantly compromise both yield and quality. To enable rapid and accurate diagnosis of cotton diseases and pests—thus supporting the development of effective control strategies and facilitating genetic breeding research—we propose a lightweight model, the Resource-efficient Cotton Network (RF-Cott-Net), alongside an open-source image dataset, CCDPHD-11, encompassing 11 disease categories. Built upon the MobileViTv2 backbone, RF-Cott-Net integrates an early exit mechanism and quantization-aware training (QAT) to enhance deployment efficiency without sacrificing accuracy. Experimental results on CCDPHD-11 demonstrate that RF-Cott-Net achieves an accuracy of 98.4%, an F1-score of 98.4%, a precision of 98.5%, and a recall of 98.3%. With only 4.9 M parameters, 310 M FLOPs, an inference time of 3.8 ms, and a storage footprint of just 4.8 MB, RF-Cott-Net delivers outstanding accuracy and real-time performance, making it highly suitable for deployment on agricultural edge devices and providing robust support for in-field automated detection of cotton diseases and pests.

## 1. Introduction

**Figure 1:**
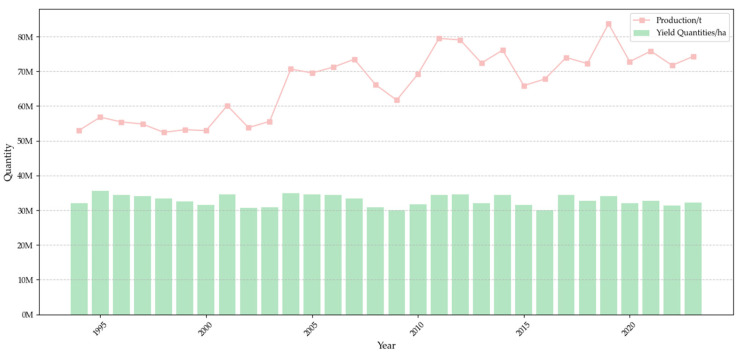
Global cotton production and yield quantities in the world from 1994 to 2023. [https://www.fao.org/faostat/zh/#data/QCL/visualize, FAO dataset, last accessed on 9 March 2025].

Cotton, a member of the genus *Gossypium* in the Malvaceae family, accounts for approximately 35% of the global demand for textile fibers [1], making it one of the most economically significant crops worldwide. As the most widely cultivated natural fiber crop, cotton serves as a vital raw material for the textile industry and plays a crucial role in the economic development and sustainable production of both fiber-producing and -consuming countries [2]. Figure 1 illustrates the global cotton production and yield in recent years. The top five cotton-producing countries are India, China, the United States, Pakistan, and Brazil [3]. While cotton yield has remained at consistently high levels and has shown an upward trend, the crop is facing frequent threats from biotic stresses such as diseases (e.g., areolate mildew [4], cotton leafroll dwarf disease [5], etc.) and pests (reniform nematode [6], southern root-knot nematode [7], etc.), which severely impact its yield and quality, resulting in substantial economic losses. Moreover, current control measures often lack precision, leading to an overreliance on chemical pesticides, high control costs, and limited effectiveness in managing cotton diseases and pests.

Effective management practices can significantly mitigate the adverse effects of diseases and pests on cotton yield and quality [8]. Achieving this requires precise monitoring and control strategies that improve efficiency while reducing costs. The foundation of precision pest and disease management lies in the accurate identification and classification of specific types of cotton diseases and pests [9]. Such accurate recognition enables researchers and producers to develop scientifically sound and cost-effective control strategies. For instance, chemical control methods (including fungicides, insecticides, and plant defense inducers) can directly suppress pathogens or activate the plant’s innate defense mechanisms [8,10,11], thereby enhancing disease resistance in cotton. However, in practical agricultural settings, it is often difficult to regulate the dosage of chemical agents. Frequent, untimely, or improper application not only elevates production costs but also poses environmental and health risks, while contributing to increased pest resistance, underscoring the urgent need for optimized management strategies [12,13]. Accurate identification supports the development and refinement of disease and pest control approaches, helping reduce cotton’s overdependence on external interventions and fostering stable, long-term natural resistance. Moreover, precise phenotypic analysis is essential for research on hostplant disease resistance improvement through genetic breeding [14], and accurate identification of plant diseases and pests forms the cornerstone of such phenotypic evaluations.

In rowers field environments, traditional methods for identifying cotton diseases and pests typically rely on continuous visual inspection by farmers, which requires a high level of domain expertise [15]. However, symptoms of infection often manifest as complex syndromes, and many pest species are small and morphologically similar [16], frequently leading to misdiagnosis and the inappropriate use of pesticides [17]. Such inaccuracies can reduce yield and quality, ultimately impacting economic returns. Moreover, manual identification is labor-intensive, inefficient, and costly [9]. To address these limitations, early research explored the application of conventional machine learning techniques for automated identification. Jenifa et al. [18] proposed a disease classification method combining median filtering, K-means clustering, and multi-class SVM to evaluate four types of cotton leaf diseases, achieving an average classification accuracy of 93.63% on their dataset. Similarly, Kurale et al. [19] integrated SVM, KNN, and artificial neural networks to classify four categories of cotton images (early blight, late blight, black rot, and healthy), attaining a classification accuracy of 91.7%. In the domain of pest detection, Ebrahimi et al. [20] utilized an SVM-based method for real-time detection of thrips (Thysanoptera) under natural lighting conditions. Fu et al. [21] employed Sentinel-2 MSI imagery in combination with ratio spectroscopy and the random forest (RF) algorithm to monitor cotton aphids, achieving an overall classification accuracy of 80%. While traditional machine learning offers advantages such as intuitive and controllable feature extraction, low computational cost, and stable performance in specific tasks, it still faces notable limitations. Its reliance on handcrafted features and domain knowledge, limited generalization and transferability, and restricted coverage in both pest/disease categories and dataset scale have significantly hindered the broader application and scalability of such approaches for cotton disease and pest identification.

With the advancement of artificial intelligence and computer vision, deep learning has opened new avenues for the automated detection and classification of cotton diseases and pests [22,23]. Owing to its capabilities in automatic feature extraction, end-to-end modeling, high recognition accuracy, strong generalization, and multimodal data fusion [9], deep learning has seen successful applications across various domains, including agriculture [24,25,26,27,28,29,30]. In the context of cotton disease and pest identification, Qiu et al. [31] developed a Transformer-based model that integrates an efficient multi-scale attention module and a stacking patch embedding module to enhance local feature learning and multi-scale information integration, achieving accurate and efficient diagnosis on both public and proprietary datasets. Similarly, Zhang et al. [32] improved the Swin Transformer architecture by incorporating residual modules, enabling precise classification of three major cotton pests: cotton aphids, cotton mirids, and cotton leaf mites. Another study systematically evaluated the performance of advanced deep learning models (including VGG16, DenseNet, MobileNet, EfficientNet, NasNet, InceptionV3, and ResNet) on cotton disease recognition tasks [33]. Due to their high accuracy and real-time capabilities, YOLO-based models have also been widely employed in this domain. Feng et al. [34] proposed an improved YOLOv8n model (LCDDN-YOLO) for detecting cotton diseases in natural field conditions. Similarly, Zhang et al. [35] developed a precise detection method for cotton diseases and pests under complex backgrounds based on the YOLOX framework. In addition, several deep learning approaches have been reported for the automated monitoring of specific cotton pests. For instance, Kakade et al. [36] employed convolutional neural networks (CNNs) and transfer learning to detect the cotton pink bollworm.

Despite notable progress in the application of deep learning for cotton disease and pest detection and classification, such models typically rely on large volumes of high-quality training data. These studies often suffer from limited dataset size and insufficient diversity, and many of the datasets used remain unpublished or inaccessible, which significantly constrains the development of deep learning-based models in this field. Moreover, under conditions of small sample sizes, limited class variety, or noisy data, deep learning models are prone to overfitting, reducing their stability and robustness in real-world applications [22]. In addition, the deployment of deep learning models in actual cotton fields is frequently hindered by limited computational resources, making it difficult to maintain high accuracy in practice. Most current research focuses primarily on improving algorithmic performance, with little attention given to making models more lightweight. As a result, these models are challenging to deploy on edge devices such as unmanned aerial vehicles (UAVs), handheld devices, mobile applications, and small unmanned ground vehicles (UGVs), greatly limiting the practical application of deep learning-based approaches for cotton disease and pest detection and classification.

To address the aforementioned challenges, we propose a lightweight model, RF-Cott-Net, for rapid and accurate detection and classification of cotton diseases and pests. The main contributions of this study are as follows:

- We collected and constructed a dataset comprising several common cotton diseases and pests by integrating the most publicly available open-source datasets with field-collected images. The resulting dataset, named the Common Cotton Diseases and Pests Huge Dataset 11 (CCDPHD-11), includes 11 categories (10 types of diseased or pest-infested cotton leaves and 1 category of healthy leaves) and contains a total of 18,953 images.
- While many datasets used in cotton disease and pest research remain unpublished, the few that are open-sourced are often limited by small sample sizes and poor diversity, which hampers research progress. In contrast, CCDPHD-11 offers a relatively large sample size and high diversity compared to existing datasets. Moreover, our dataset will be made publicly available to promote further research in automated cotton disease and pest diagnosis.
- We propose the Resource-efficient Cotton Network (RF-Cott-Net), a lightweight deep learning model based on the MobileViTv2 architecture. By integrating quantization-aware training (QAT) and an early exit mechanism, RF-Cott-Net achieves a well-balanced trade-off between model compactness and performance. Experimental results demonstrate the model’s strong effectiveness in cotton disease and pest diagnostic tasks.
- On the proposed CCDPHD-11 dataset, our method achieves an accuracy of 98.4% across 11 classes, including healthy cotton leaves. The model size has a size of just 4.8 MB, with a 4.9 M parameter count, 310 M FLOPs, and inference latency of only 3.8 ms. These results demonstrate that RF-Cott-Net offers a practical solution for efficient, rapid, and low-cost on-device cotton disease and pest diagnosis.

Overall, this study aims to address the challenges of insufficient data volume and limited sample diversity in cotton disease and pest diagnosis. By introducing an innovative lightweight deep learning model, we enhance the level of automation in disease and pest identification, offering valuable insights for the future development of precision agriculture. The remainder of this paper is organized as follows: Section 2 outlines the methods and materials used in this study, including the construction of the dataset and the proposed model; Section 3 presents the experimental design; Section 4 details and analyzes the experimental results; Section 5 discusses the strengths and limitations of the study as well as potential directions for future improvement; and Section 6 concludes the paper.

## 2. Methods and Materials

### 2.1. Dataset

**Figure 2:**
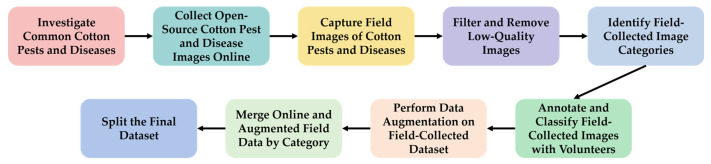
Workflow of constructing our proposed cotton pest and disease dataset (CCDPHD-11).

The dataset in this study was constructed by following nine main steps [16]: (1) investigating common categories of cotton diseases and pests; (2) collecting publicly available open-source datasets from online sources; (3) capturing field images of various cotton diseases and pests; (4) filtering and removing low-quality images; (5) identifying the categories of field-collected images; (6) recruiting volunteers to annotate and classify the images, thereby forming the raw field-collected dataset; (7) applying data augmentation to the field-collected images to generate an enhanced dataset; (8) merging the online datasets with the augmented field-collected dataset by category to form the final dataset; and (9) dividing the final dataset into appropriate subsets. The overall dataset construction workflow is illustrated in Figure 2.

**Figure 3:**
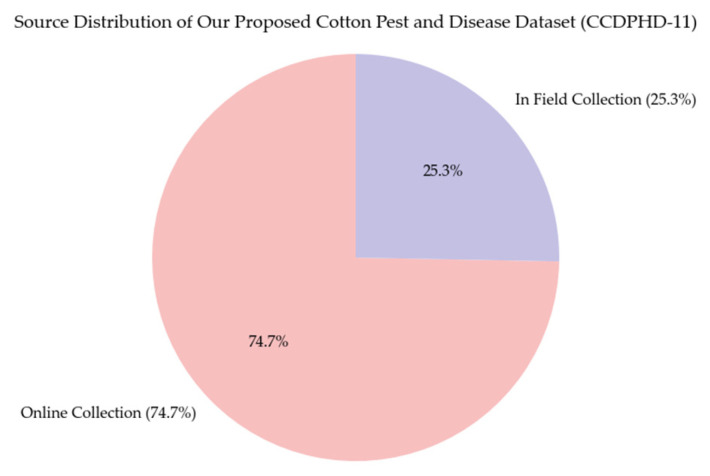
Source distribution of our proposed cotton pest and disease dataset (CCDPHD-11).

In the final dataset constructed, 74.8% of the images were sourced from other open-source datasets, while 25.2% were obtained through field collection, as illustrated in Figure 3. Prior to collecting online open-source datasets, an extensive search was conducted on major platforms such as Kaggle and GitHub to identify publicly available datasets related to cotton diseases and pests. In addition, Google Scholar was searched using the keywords “cotton”, “disease”, and “pest” to locate open-source datasets cited in the published literature. During the process of online data acquisition, several criteria were considered for initial screening, including the source and scale of the dataset, the credibility and reputation of the dataset or platform, the number of cotton disease and pest categories included, and the consistency and accuracy of annotations. Despite the preliminary screening, some selected datasets still contained low-quality or irrelevant data. To ensure the scientific rigor of this study, all collected open-source datasets were further cleaned by removing poor-quality or unrelated images, as well as duplicate entries across different sources. As a result, a total of 14,177 high-quality images of cotton diseases and pests were retained from online sources.

**Figure 4:**
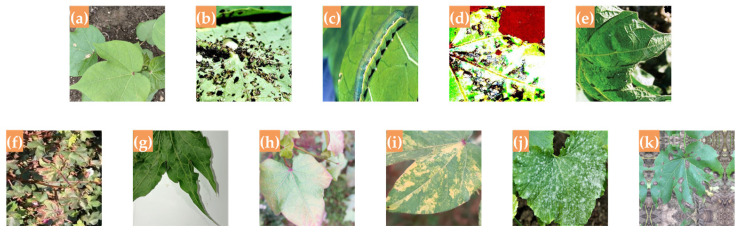
Samples of cotton diseases and pests in CCDPHD-11: (**a**) healthy cotton; (**b**) aphids; (**c**) army worm; (**d**) bacterial blight; (**e**) cotton curl virus; (**f**) fusarium wilt; (**g**) herbicide growth damage; (**h**) leaf redding; (**i**) leaf variegation; (**j**) powdery mildew; (**k**) target spot.

**Figure 5:**
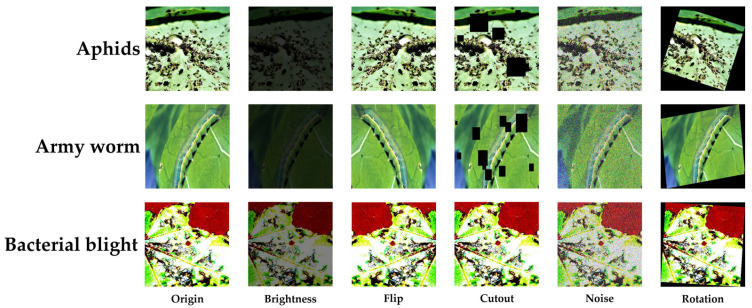
Some samples of our in-field collected data after augmentation in CCDPHD-11 (including original images).

For the field-collected dataset, images of several types of cotton diseases and pests were captured at two sites: the Shangzhuang Experimental Station (116.185571° E, 40.137692° N) and the Zhuozhou Teaching and Experimental Field (116.0722° E, 39.4706° N) of China Agricultural University. A total of approximately 1200 raw images were initially collected. After acquiring relevant knowledge of cotton diseases and pests through online learning, five volunteers, together with three of the authors of this study, collaboratively screened the candidate images. Due to motion-induced blur and the inclusion of irrelevant content during image capture, blurry and unrelated images were removed, resulting in 796 usable field-collected images. These images were then uniformly converted to JPEG format and categorized accordingly. To enhance dataset diversity and improve model robustness, we applied various data augmentation techniques, including random brightness adjustment, flipping, random masking (with 1 to 20 randomly generated masks of varying sizes), Gaussian noise addition, and random rotation. Through this process, a total of 4776 augmented images were obtained. Finally, the online-collected images were merged with the augmented field-collected images to form a unified dataset consisting of 18,953 images across 11 categories (including one category of healthy cotton leaves), with a total dataset size of 2.67 GB, named the Common Cotton Diseases and Pests Huge Dataset (CCDPHD-11). The dataset was then split into a training set (15,159 images) and a test set (3794 images) at a ratio of 8:2. Sample images from CCDPHD-11 are shown in Figure 4; examples before and after augmentation of field-collected images are presented in Figure 5; and the number of images per category is summarized in Table 1.

### 2.2. MobileViTv2

In cotton disease and pest recognition tasks, models must simultaneously meet multiple requirements—including high recognition accuracy, lightweight architecture, and ease of deployment—to enable practical application and large-scale adoption in real-world cotton fields. MobileViT, first introduced by Apple in 2021 [37], is a lightweight vision architecture that combines the strengths of CNNs and Vision Transformers (ViTs). It offers notable improvements in both representational power and computational efficiency. Specifically, MobileViT leverages convolutional layers to extract local detail features while incorporating Transformer mechanisms for global feature modeling. This design enables the model to achieve strong image recognition performance while maintaining low parameter count and computational cost, providing a promising solution for agricultural image analysis tasks. Building upon MobileViTv1, Mehta et al. [38] later proposed MobileViTv2, which delivers further enhancements in accuracy, efficiency, generalization ability, and deployment flexibility.

Compared to MobileViTv1, the MobileViTv2 model features significant architectural optimizations. By introducing an improved MobileViT Block and a decoupled attention mechanism, it enhances feature interaction and representation while reducing redundant computation and improving inference speed. Specifically, MobileViTv2 adopts a separable self-attention structure to decouple and optimize the process of global context modeling. This mechanism computes the global context vector cv using the constraint formulation shown in Equation (1).

(1) $$cv=\sum_{i=1}^{k}cs\left(i\right)\cdot x_{K}\left(i\right)$$

Here, $cs\left(i\right)$ denotes the contextual weight at each position, $x_{K}\left(i\right)$ represents the output of the key branch, $cv\in {\mathbb{R}}^{d}$ denotes the extracted context vector.

Subsequently, the context vector is fused with the output of the value branch after activation, yielding the final output y, as shown in Equation (2).

(2) $$y=\left(cv*\mathrm{ReLU}\left(xW_{V}\right)\right)W_{O}$$

Here, ∗ denotes broadcast element-wise multiplication, and $W_{V}$ and $W_{O}$ are linear transformation matrices. Compared to the conventional multi-head attention mechanism, this design significantly reduces computational complexity, making it more suitable for deployment on resource-constrained agricultural devices while maintaining model performance.

In addition, MobileViTv2 significantly improves image classification performance while maintaining a compact architecture, making it particularly well suited for agricultural scenarios with limited computational resources, such as in-field applications. Compared to the more recent MobileViTv3 [39], MobileViTv2 features lower architectural complexity and a reduced training barrier, offering greater practicality and controllability in tasks like cotton disease and pest recognition, where training samples are relatively limited. Therefore, in terms of accuracy, efficiency, deployability, and debugging cost, MobileViTv2 serves as the most practically suitable baseline model to date.

In addition to the MobileViT series, conventional classification models such as ResNet, VGG, EfficientNet, and MobileNetV3 are also commonly used as baseline architectures. Although ResNet [40] offers strong deep feature extraction capabilities, its large number of parameters and slow inference speed make it unsuitable for deployment on edge computing devices. The VGG network [41] has an even larger architecture and lacks modular design, resulting in high training and inference costs. While EfficientNet [42] demonstrates certain advantages in model lightweighting, it heavily depends on specific training strategies and shows limitations in feature representation for certain tasks. MobileNetV3 [43], which relies heavily on depthwise separable convolutions and NAS optimization, suffers from limited receptive fields and struggles with complex agricultural backgrounds and subtle inter-class variations. In contrast, MobileViTv2 is better suited to efficient multi-object recognition and classification under resource-constrained conditions, offering greater adaptability to domain-specific applications.

In summary, MobileViTv2 is an ideal model that balances performance and deployment efficiency, making it particularly suitable for tasks such as cotton disease and pest detection, which are sensitive to resource constraints and demand real-time processing. Therefore, this study adopts MobileViTv2 as the baseline model, laying the foundation for subsequent improvements and lightweight optimization.

### 2.3. Early Exit Mechanism

To enhance the inference efficiency of the cotton disease and pest detection and classification model while maintaining accuracy, this study incorporates an early exit mechanism [44] during model construction. The core idea of this mechanism is that not all input samples require deep-layer processing to achieve accurate predictions; for easily distinguishable samples, inference can be terminated at earlier network layers, thereby reducing computational resource consumption.

Specifically, we insert lightweight classification modules (referred to as early-exit stages) after several intermediate layers of the model, each capable of independently generating a prediction. During inference, the model progressively processes the input image from the initial layer and at each early exit stage computes a probability distribution over the predicted classes. If the output entropy at a given stage falls below a predefined confidence threshold, indicating sufficient certainty in the prediction, the result is returned directly, and subsequent layers are bypassed. If the threshold is not met, the input continues to propagate through deeper layers until an exit condition is satisfied or the final layer is reached.

The early exit mechanism offers several notable advantages [45,46]: (1) it significantly reduces inference latency and improves system response speed; (2) it exhibits sample-adaptive behavior by dynamically selecting inference paths based on input complexity; (3) it enhances model flexibility and resource adaptability, making it more suitable for deployment on edge devices; and (4) it introduces a regularization effect during training, which helps improve the model’s generalization capability.

Moreover, the early exit mechanism is particularly well suited for cotton disease and pest detection and classification tasks. On one hand, the visual complexity of different diseases and pests varies significantly; this mechanism allows rapid processing of samples with distinctive features, thereby conserving computational resources. On the other hand, agricultural applications often require large-scale image processing and edge deployment. The early exit mechanism enhances system processing efficiency and energy adaptability while maintaining accuracy, thus providing strong support for practical in-field deployment.

### 2.4. Quantization-Aware Training

Quantization-aware training (QAT) [47] was first introduced by Google in 2018 with the goal of enabling efficient inference of deep neural networks under low-precision integer formats (e.g., INT8) while minimizing accuracy degradation. This approach simulates quantization errors during the training process, allowing the model to adapt to the low-precision computational environment expected during inference.

Compared with traditional post-training quantization (PTQ) [48,49], QAT offers significant advantages. While PTQ is computationally straightforward, it often suffers from performance degradation in tasks involving complex data distributions or requiring high accuracy. In contrast, QAT models and optimizes quantization errors during training, enabling the quantized model to maintain performance close to that of its full-precision counterpart during deployment. Given the high demands on feature discriminability and classification accuracy in cotton disease and pest detection under open-field conditions, QAT’s ability to account for quantization error during training makes it particularly well suited for this application.

**Figure 6:**
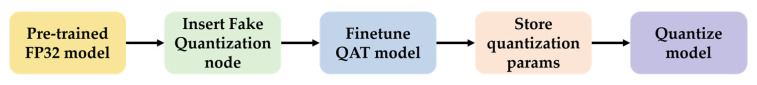
Workflow of quantization-aware training (QAT).

To provide a clearer illustration of the QAT implementation process used in this study, Figure 6 presents the five key steps involved in INT8 quantization: (1) obtaining a baseline model with FP32 precision trained on the dataset; (2) inserting fake quantization nodes into the model; (3) fine-tuning the model with the inserted fake quantization nodes; (4) preserving the quantization parameters of each layer during training; and (5) exporting the final INT8 quantized model using the saved parameters for deployment within the inference framework.

In this study, we incorporated the QAT strategy into the MobileViTv2 baseline to develop a more lightweight and high-performance model for cotton disease and pest detection and classification. Through QAT optimization, the model achieves a smaller parameter size and reduced floating point computation requirements, which significantly lowers memory usage and power consumption when deployed on edge devices or mobile terminals while also improving response speed. Moreover, since QAT preserves the representational capacity of the original floating point model during training, it enhances inference efficiency without compromising accuracy.

In summary, QAT serves as a deployment-oriented deep model compression technique that not only effectively reduces model size and computational complexity but also maintains recognition accuracy. It meets the efficiency and practicality demands of intelligent agricultural devices, providing essential support for building lightweight models for cotton disease and pest detection and classification.

### 2.5. Proposed Model (RF-Cott-Net)

#### 2.5.1. Optimization in the Training Process

Based on the MobileViTv2 architecture, this study proposes the Resource-efficient Cotton Network (RF-Cott-Net) model for cotton disease and pest image diagnosis. The backbone integrates the lightweight convolutional module MobileNetV2 block (MV2B) with the Transformer-based MobileViT block, thereby preserving local texture information while enabling global feature modeling. The model initially extracts low-level features using a 3 × 3 convolution, followed by a sequence of MV2B modules and deep MobileViT blocks to capture multi-scale semantic information.

To enhance the representational capacity and learning depth during training, we introduce a multi-branch early exit mechanism. Three auxiliary classification heads are attached after the main MobileViT blocks, forming early exit stages 1, 2, and 3. These auxiliary branches serve as intermediate supervision signals during training, effectively promoting feature learning in the shallow layers and improving overall training performance. Each early exit stage is associated with its own loss function, and parameters are updated accordingly during training, as defined in Equation (3).

(3) $$L_{i}\left(D;\theta \right)=\frac{1}{\left|D\right|}\sum_{\left(x,y\right)\in D}H\left(y,f_{i}\left(x;\theta \right)\right)$$

where D represents the training set; θ is the collection of all parameters; $\left(x,y\right)$ denotes the feature–label pair of a sample; H represents the cross-entropy loss function; and $f_{i}$ is the output of the $i^{th}$ stage.

Compared to conventional lightweight models that rely solely on a single final output, this mechanism significantly enhances the semantic representation capability of intermediate layers. In particular, when dealing with samples exhibiting early-stage lesions, sparsely distributed pests, or unclear image boundaries, the early exit strategy enables the model to receive multi-level discriminative guidance from different network depths, thereby improving overall generalization performance.

#### 2.5.2. Optimization in Inference Process

During inference, to improve computational efficiency and resource utilization, RF-Cott-Net adopts a confidence threshold–based dynamic early exit mechanism. Specifically, three early exit branches (early exit 1, 2, and 3) are placed after MobileViT blocks at varying depths, each associated with a predefined confidence threshold (0.6, 0.7, and 0.8, respectively).

When the model’s prediction confidence at any early exit stage exceeds the corresponding threshold, inference is terminated, and the prediction is output directly. For example, if the confidence at stage 1 surpasses 0.6, the model halts further computation and returns the result, avoiding the more computationally intensive subsequent modules. The confidence-based dynamic early exit mechanism is detailed in Algorithm 1. This mechanism offers several advantages during inference: (1) for images with distinct features and prominent manifestations of cotton diseases or pests, the model often achieves sufficient confidence at stage 1 or 2, allowing for early termination and reduced inference time; (2) for images with complex backgrounds or subtle features, the model proceeds to stage 3 or the final output layer to ensure accuracy; (3) the strategy is highly adaptive, dynamically adjusting the inference path based on image complexity, thereby significantly improving the model’s practicality for edge deployment.
**Algorithm 1** RF-Cott-Net Inference (Input: *X*)**for** *i* = 1 to *n* **do**  *z_i_* = *f_i_* (*X*; *θ*)  **if** entropy (*z_i_*) < S **then**   **return** *z_i_*  **end if**
**end for****return** *z_n_*

#### 2.5.3. Structural Compression and Deployment Adaptability Enhancement

To further reduce model size and computational load, this study incorporates the quantization-aware training (QAT) strategy, compressing the model from full-precision (FP32) to low-bitwidth (INT8) format to meet practical deployment requirements in real-world cotton field environments. Unlike traditional post-training quantization (PTQ), QAT introduces simulated quantization operators during training, enabling both weights and activations to be processed with low precision during forward and backward propagation. This approach effectively mitigates the accuracy degradation typically caused by quantization and is particularly well suited for complex architectures involving Transformer-based MobileViT blocks.

**Figure 7:**
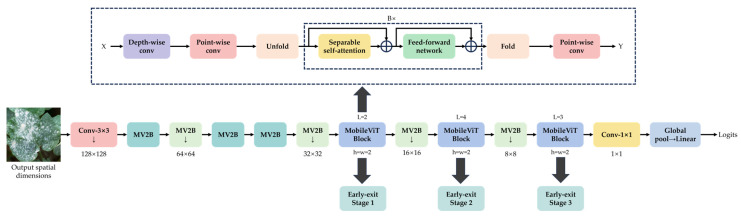
Overall structural diagram of our proposed model (RF-Cott-Net). Note: MobileNetV2 blocks that perform down-sampling are marked with ↓.

In addition, when combined with the early exit mechanism, QAT not only reduces the computational and memory load along each inference path but also enhances the low-precision robustness of all early exit branches. Even under the INT8 format, RF-Cott-Net maintains stable confidence-based decision logic across all three early exit stages, ensuring the consistency and reliability of prediction outputs. The QAT strategy significantly compresses model size and lowers power consumption, enabling efficient inference performance on lightweight platforms such as Jetson Nano and Raspberry Pi, thereby offering strong support for practical deployment in cotton fields. The overall architecture of the proposed RF-Cott-Net model is illustrated in Figure 7.

## 3. Experiments and Evaluation Metrics

### 3.1. Model Training Device and Parameters’ Setup

In this study, model training was conducted on an integrated lift-configured system equipped with an NVIDIA V100 GPU (32 GB memory) (NVIDIA, Santa Clara, CA, USA). The computational resources included a 6-core Intel^®^ Xeon^®^ Gold 6130 CPU (2.10 GHz) and 25 GB of RAM (Intel, Santa Clara, CA, USA). During training, the batch size was set to 128 and the learning rate to 5 × 10^−5^, and the AdamW optimizer was employed to ensure stable convergence and high-accuracy classification performance.

### 3.2. Model Evaluation Experiment

#### 3.2.1. Baseline Models for Comparative Evaluation

To comprehensively validate the effectiveness of the proposed lightweight cotton disease and pest detection and classification model (RF-Cott-Net), we compared it against several mainstream lightweight baseline models. These baselines fall into two categories: lightweight Transformer/hybrid architectures and conventional CNN models.

For the lightweight Transformer/hybrid models, we selected MobileViTv1-XS, DeiT-Tiny, and EdgeViT-XXS. MobileViTv1-XS [37] is a streamlined version of the original MobileViT, combining convolutional operations with Transformer modules to achieve strong global modeling capability while maintaining low computational complexity. DeiT-Tiny [50], a pure Transformer-based model, employs self-attention mechanisms for efficient visual feature extraction and is well suited for training scenarios with limited data. EdgeViT-XXS [51] is specifically optimized for edge devices, leveraging token pruning and efficient architectural design to balance model accuracy and computational cost.

For conventional CNN models, we selected MobileNetV3-Large, EfficientNet-B0, and ResNet-18 for comparison. MobileNetV3-Large [43] integrates depthwise separable convolutions with SE modules and employs automated architecture search to optimize its structure, making it widely used in mobile vision applications. EfficientNet-B0 [42] adopts a compound scaling strategy to enhance model performance across multiple dimensions, offering high parameter efficiency. ResNet-18 [40], as a classic residual network, has a relatively larger number of parameters but remains representative due to its robustness and stability, making it a suitable baseline among traditional CNNs in this study.

By incorporating these diverse baseline models for comparative experiments, we are able to comprehensively evaluate the overall advantages of RF-Cott-Net in terms of accuracy, parameter count, and computational cost for lightweight and task-specific cotton disease and pest detection and classification.

#### 3.2.2. Evaluation Metrics

To evaluate the effectiveness of the proposed RF-Cott-Net model for cotton disease and pest detection and classification, this study adopts a set of widely used performance metrics from the field of object detection [52]. The evaluation metrics include precision, accuracy, recall, and F1-score (F1). Precision measures the correctness of predictions for a specific class; accuracy reflects the proportion of correctly predicted samples among all samples and is suitable for assessing overall model performance on balanced datasets; recall quantifies the proportion of actual positive samples that are correctly identified by the model; and F1-score, the harmonic mean of precision and recall, provides a comprehensive assessment of model performance under class-imbalanced conditions. The definitions of these metrics are presented in Equations (4)–(7).

(4) $$\mathrm{Precision}=\frac{tp}{tp+fp}$$

(5) $$\mathrm{Accuracy}=\frac{tp+tn}{tp+fp+tn+fn}$$

(6) $$\mathrm{Recall}=\frac{tp}{tp+fn}$$

(7) $$F1\text{-Score}=\frac{2\times \mathrm{Precision}\times \mathrm{Recall}}{\mathrm{Precision}+\mathrm{Recall}}$$

where *tp* denotes true positives, the number of samples correctly predicted to belong to a specific class; *fp* denotes false positives, the number of samples incorrectly predicted to belong to a specific class; *tp* denotes true negatives, the number of samples correctly predicted to not belong to a specific class; and *fn* denotes false negatives, the number of samples that belong to a specific class but were incorrectly predicted to belong to another class.

### 3.3. Ablation Experiment

To comprehensively assess the effectiveness and performance of the proposed RF-Cott-Net model for cotton disease and pest detection and classification, in this study, we designed and conducted an ablation study. Specifically, we constructed and compared three model variants—(1) RF-Cott-Net (MobileViTv2 + QAT + early-exit mechanism), (2) MobileViTv2 + early-exit mechanism, and (3) MobileViTv2 + QAT—in order to investigate the individual contributions of the QAT and early exit mechanism strategies in terms of accuracy and efficiency.

The ablation study helps clarify the impact of each lightweight strategy on the final model performance and verifies the effectiveness of different combinations. By comparing multiple evaluation metrics (including parameter count (Params), floating point operations (FLOPs), inference latency, accuracy, F1-score, precision, and recall), we provide a holistic evaluation of RF-Cott-Net from the perspectives of model compression, computational efficiency, and recognition performance. The results also offer practical insights for deploying intelligent cotton disease and pest recognition systems in resource-constrained environments.

## 4. Results

### 4.1. Results of Model Evaluation Experiment

To comprehensively evaluate the performance of the proposed cotton disease and pest detection and classification model, we designed and conducted a series of validation experiments involving representative models from mainstream lightweight CNNs, ViT (hybrid) architectures, and pure Transformer frameworks. All models were trained and tested under identical conditions using the same dataset (CCDPHD-11) and training strategy. The input image sizes were uniformly resized to either 224 × 224 or 256 × 256, and the batch size was set to 64 to ensure fairness and consistency in comparative evaluation.

Table 2 presents the performance of all models across key metrics, including parameter count (Params), floating point operations (FLOPs), storage efficiency, inference latency, accuracy, F1-score (F1), precision, and recall. The results demonstrate that the proposed RF-Cott-Net model achieves outstanding performance across multiple critical indicators. With only 4.9 M parameters and 310 M FLOPs, RF-Cott-Net attains 98.4% accuracy, 98.4% F1-score, 98.5% precision, and 98.3% recall, while maintaining the lowest inference latency among all models at just 3.8 ms. Additionally, the model size is only 4.8 MB, highlighting its high efficiency and practical value for real-world applications in cotton disease and pest detection and classification.

In comparison, although ResNet-18 (conventional CNN model) achieves a relatively high accuracy of 97.9%, it has a large parameter count of 11.2 M, FLOPs of 2.4 G, and a storage size of 42.7 MB, making it unsuitable for deployment on edge devices. MobileNetV3, despite its small parameter size of only 2.4 M, exhibits the slowest inference latency among all models at 18.5 ms, rendering it inadequate for time-sensitive agricultural applications. EfficientNet-B0 demonstrates relatively balanced performance but still falls short of the proposed RF-Cott-Net. Within the hybrid architecture category, we evaluated both MobileViTv2-0.75 and MobileViTv2-1.0. The MobileViTv2-0.75 version, constructed with a 0.75 width scaling factor, has a model size of 9.6 MB and further reduces parameters (2.5 M) and FLOPs (1.0 G), but shows a noticeable drop in accuracy to 96.6%. The standard-width MobileViTv2-1.0 achieves 98.7% accuracy with 4.3 M parameters and 1.9 G FLOPs, but its inference latency of 10.3 ms limits its lightweight and real-time applicability. By contrast, RF-Cott-Net delivers comparable performance with only 4.8 MB in storage size and 310 M FLOPs and achieves the fastest inference latency at 3.8 ms. As for the pure Transformer models, DeiT-Tiny and EdgeViT-XXS achieve lower accuracies of 92.1% and 94.2%, respectively, indicating their limited adaptability to complex, texture-inhomogeneous agricultural images.

Overall, the proposed RF-Cott-Net model achieves the most favorable balance of performance, maintaining high accuracy while delivering the lowest inference latency and a compact model size. These characteristics make it particularly well suited for deployment on resource-constrained agricultural edge devices.

### 4.2. Results of Alation Experiment

To further validate the contribution of each key strategy within the proposed RF-Cott-Net model, we conducted ablation experiments to investigate the impact of the two lightweight strategies, the early exit mechanism and quantization-aware training (QAT), on overall model performance. The experimental results are presented in Table 3.

Specifically, based on the same MobileViTv2 backbone, we independently incorporated the early exit mechanism and QAT strategy for evaluation. The results show that MobileViTv2 + early exit mechanism achieved 98.5% Accuracy with 4.4 M parameters and 1.5 G FLOPs, while also maintaining high F1-score, precision, and recall. This indicates that the early exit mechanism can enhance the flexibility of the inference path without compromising performance. However, its inference latency reached 8.7 ms, which is still higher than that of RF-Cott-Net, revealing a slight disadvantage in terms of real-time responsiveness. On the other hand, MobileViTv2 + QAT significantly reduced computational cost (470 M FLOPs) and inference latency (5.2 ms) without sacrificing classification performance, demonstrating that the QAT strategy is highly beneficial for deployment in resource-constrained agricultural scenarios.

The final integrated model, RF-Cott-Net, achieved a high accuracy of 98.4% while reducing FLOPs to 310 M, maintaining an inference latency of only 3.8 ms and a parameter count of just 4.9 M. These results highlight the model’s well-balanced trade-off between lightweight design and classification performance. The ablation study confirms the synergistic benefits of the joint optimization strategy (early exit mechanism combined with QAT), demonstrating enhanced deployability and strong potential for practical application in real-world cotton field scenarios.

## 5. Discussion

### 5.1. Advantages

Although several cotton disease and pest datasets have been developed in previous studies, most still suffer from limited sample sizes and insufficient data diversity, as shown in Table 4. For instance, Bishshash et al. [53] collected 2137 raw images and created a dataset comprising 7000 samples covering seven common cotton diseases. However, this dataset exhibits limited variability in lighting conditions and lacks representation of pest-related symptoms. In contrast, the CCDPHD-11 dataset constructed in this study combines field-collected and publicly sourced data, encompassing a wider range of cotton diseases and pests, thereby offering greater representativeness and generalization capacity. Furthermore, our dataset will be made publicly available to the research community, with the aim of advancing research in automated cotton disease and pest diagnosis.

Many models for cotton disease and pest detection prioritize accuracy improvements while overlooking the importance of model lightweighting and deployability. These studies often adopt complex model architectures to boost detection performance, resulting in high computational costs and significant inference latency, which severely limits their applicability in real-world cotton field environments.

In contrast, the lightweight RF-Cott-Net model can effectively address these issues by balancing accuracy with computational efficiency. By incorporating QAT and the early exit mechanism, our model significantly reduces parameter count and FLOPs while maintaining strong performance, thereby enabling the potential for real-time deployment in agricultural edge devices.

### 5.2. Disadvantages

While RF-Cott-Net demonstrates strong overall performance, several limitations remain to be addressed. First, the current CCDPHD-11 dataset primarily consists of images depicting severely infected cotton plants, which may limit the model’s effectiveness in early warning and classification of mild or early-stage symptoms. However, early detection plays a critical role in facilitating timely field interventions and adjusting pest and disease control strategies. This study primarily focuses on the identification and classification of already affected plants, without exploring early-stage warning capabilities. Second, while the model achieves high efficiency, further reducing the parameter count while maintaining robustness remains a challenge. Moreover, the model is currently validated at the algorithmic level and has yet to be tested under real-world cotton field conditions. As such, its performance may be affected by practical challenges such as variable lighting, occlusion, and noise commonly present in field environments.

### 5.3. Future Perspectives

To address the aforementioned limitations and enhance the practical applicability of the model, future research may proceed in the following directions.

- **Data expansion through synthetic generation:** Future studies may leverage generative adversarial networks and related techniques to synthesize early-stage or rare disease and pest images [54], thereby enriching the dataset and improving the model’s ability to recognize mild infections.
- **Cross-domain adaptation:** Given the substantial environmental variability across different cotton-growing regions, future work could explore domain adaptation and transfer learning strategies to improve the model’s robustness and adaptability under diverse geographical and environmental conditions.
- **Platform migration and edge deployment:** RF-Cott-Net may be further validated through deployment in UAVs, handheld devices, mobile applications, or compact UGVs. These platforms impose stringent constraints on inference speed and energy consumption, and the lightweight design proposed in this study lays the groundwork for practical deployment in such scenarios.
- **Moving towards a full pipeline for cotton disease and pest control:** Future research could expand RF-Cott-Net into a comprehensive management system that integrates early warning, risk assessment, and decision support for cotton disease and pest control, thereby enhancing its utility and level of intelligence.
- **Multimodal data fusion for comprehensive diagnosis:** Future research could incorporate multimodal data sources—such as visible light, hyperspectral, thermal, and meteorological data—to enhance the comprehensiveness and precision of cotton disease and pest detection. By fusing heterogeneous data modalities, the system could better capture subtle physiological and environmental indicators, thereby improving early-stage diagnosis, disease differentiation, and stress factor attribution [55].

In summary, while many existing studies emphasize accuracy at the expense of lightweight design and deployability, this study simultaneously addresses both performance and feasibility, providing strong support for the scalable and practical implementation of intelligent cotton disease and pest diagnosis.

## 6. Conclusions

In this study, we proposed a lightweight deep learning model, RF-Cott-Net, for accurate and efficient diagnosis of cotton diseases and pests. Built upon the MobileViTv2 backbone and incorporating an early exit mechanism and quantization-aware training (QAT), the model achieves a strong balance between performance and deployability. Evaluated on the self-constructed CCDPHD-11 dataset (comprising 11 classes), RF-Cott-Net reached an accuracy of 98.4%, an F1-score of 98.4%, precision of 98.5%, and recall of 98.3%. It also demonstrated high efficiency with a model size of 4.8 MB, 4.9 M parameters, 310 M FLOPs, and an inference latency of 3.8 ms. These results indicate the model’s potential for real-time deployment in edge devices, providing a reliable foundation for automated in-field cotton disease and pest detection.

## Figures and Tables

**Table 1:** Sample size of each class of cotton pests and diseases in the CCDPHD-11 dataset.

Class	Training Set	Test Set	Total
Healthy	5155	1289	6444
Aphids	1265	317	1582
Amy worm	1088	272	1360
Bacterial blight	1981	496	2477
Cotton curl virus	2024	506	2530
Fusarium wilt	704	177	881
Herbicide growth damage	224	56	280
Leaf redding	462	116	578
Leaf variegation	92	24	116
Powdery mildew	1080	270	1350
Target spot	1084	271	1355
Total	15,159	3794	18,953

**Table 2:** Performance comparison between the proposed model and mainstream lightweight models.

Model	Type	Storage Efficiency	Params	FLOPs	Latency (ms)	Accuracy (%)	F1 (%)	Precision (%)	Recall (%)
EfficientNet-B0	CNN	15.6 MB	3.8 M	495 M	10.6	97.4	97.4	97.5	97.3
MobileNetV3	CNN	16.3 MB	4.2 M	280 M	18.5	98.1	98.1	98.2	98.0
ResNet-18	CNN	42.7 MB	11.2 M	2.4 G	3.4	97.9	97.9	98.0	97.8
MobileViT-S	Hybrid	19 MB	4.9 M	1.9 G	14.5	98.1	98.1	98.2	98.0
MobileViTv2-0.75	Hybrid	9.6 MB	2.5 M	1.0 G	9.8	96.6	96.6	96.8	96.4
MobileViTv2-1.0	Hybrid	16.9 MB	4.3 M	1.9 G	10.3	98.7	98.7	98.8	98.6
DeiT-Tiny	Transformer	21.2 MB	5.5 M	1.1 G	12.3	92.1	92.1	92.3	92.0
EdgeViT-XXS	Transformer	14.8 MB	4.1 M	0.6 G	9.5	94.2	94.2	94.3	94.1
**RF-Cott-Net**	Hybrid	4.8 MB	4.9 M	310 M	3.8	98.4	98.4	98.5	98.3

**Table 3:** Results of ablation experiments using different strategies.

Model	Params	FLOPs	Latency (ms)	Accuracy (%)	F1 (%)	Precision (%)	Recall (%)
MobileViTv2 + early exit	4.4 M	1.5 G	8.7	98.5	98.5	98.6	98.4
MobileViTv2 + QAT	4.3 M	470 M	5.2	98.5	98.5	98.6	98.4
**RF-Cott-Net**	4.9 M	310 M	3.8	98.4	98.4	98.5	98.3

**Table 4:** Comparison with existing datasets related to cotton diseases and pests.

Dataset	Class	Ava	Samp	Ave	Year	Reference
NA	4	No	240	60	2019	[18]
CottonInsect	6	Yes	3225	537.5	2021	[31]
NA	3	No	2705	901.6	2024	[32]
NA	8	No	6712	839	2025	[34]
NA	5	No	5760	1152	2022	[35]
SAR-CLD-2024	7	Yes	7000	1000	2024	[53]
**CCDPHD-11**	11	Yes	18,953	1723	2025	\

**Note:** “NA” represents that the dataset has no specific name. “Class” denotes the class number of the dataset. “Ava” indicates if the dataset is open access. “Samp” is the sample size of the dataset. “Ave” represents the average number of samples per class.

## Data Availability

The proposed CCDPHD-11 dataset can be found online (https://github.com/SweefongWong/CCDPHD-11-Dataset, accessed on 9 March 2025).

## References

[1] Huang G., Huang J.-Q., Chen X.-Y., Zhu Y.-X. (2021). Recent Advances and Future Perspectives in Cotton Research. Annu. Rev. Plant Biol..

[2] Scarpin G.J., Bhattarai A., Hand L.C., Snider J.L., Roberts P.M., Bastos L.M. (2025). Cotton Lint Yield and Quality Variability in Georgia, USA: Understanding Genotypic and Environmental Interactions. Field Crops Res..

[3] Khan M.A., Wahid A., Ahmad M., Tahir M.T., Ahmed M., Ahmad S., Hasanuzzaman M., Ahmad S., Hasanuzzaman M. (2020). World Cotton Production and Consumption: An Overview. Cotton Production and Uses: Agronomy, Crop Protection, and Postharvest Technologies.

[4] Jimenez Madrid A.M., Munoz G., Wilkerson T., Chee P.W., Kemerait R. (2025). Identification of Ramulariopsis Pseudoglycines Causing Areolate Mildew of Cotton in Georgia and First Detection of QoI Resistant Isolates in the United States. Plant Dis..

[5] Edula S.R., Bag S., Milner H., Kumar M., Suassuna N.D., Chee P.W., Kemerait R.C., Hand L.C., Snider J.L., Srinivasan R. (2023). Cotton Leafroll Dwarf Disease: An Enigmatic Viral Disease in Cotton. Mol. Plant Pathol..

[6] Wubben M.J., Khanal S., Gaudin A.G., Callahan F.E., McCarty J.C., Jenkins J.N., Nichols R.L., Chee P.W. (2025). Transcriptome Profiling and RNA-Seq SNP Analysis of Reniform Nematode (*Rotylenchulus reniformis*) Resistant Cotton (*Gossypium hirsutum*) Identifies Activated Defense Pathways and Candidate Resistance Genes. Front. Plant Sci..

[7] Khanal S., Kumar P., da Silva M.B., Singh R., Suassuna N., Jones D.C., Davis R.F., Chee P.W. (2025). Time-Course RNA-Seq Analysis of Upland Cotton (*Gossypium hirsutum* L.) Responses to Southern Root-Knot Nematode (*Meloidogyne incognita*) during Compatible and Incompatible Interactions. BMC Genom..

[8] Ellsworth P.C., Fournier A. (2025). Theory versus Practice: Are Insecticide Mixtures in Arizona Cotton Used for Resistance Management?. Pest Manag. Sci..

[9] Qin Y.-M., Tu Y.-H., Li T., Ni Y., Wang R.-F., Wang H. (2025). Deep Learning for Sustainable Agriculture: A Systematic Review on Applications in Lettuce Cultivation. Sustainability.

[10] Qiu P., Zheng B., Yuan H., Yang Z., Lindsey K., Wang Y., Ming Y., Zhang L., Hu Q., Shaban M. (2024). The Elicitor VP2 from Verticillium Dahliae Triggers Defence Response in Cotton. Plant Biotechnol. J..

[11] Rani M., Murali-Baskaran R.K. (2025). Synthetic Elicitors-Induced Defense in Crops against Herbivory: A Review. Plant Sci..

[12] Liu E.M., Huang J. (2013). Risk Preferences and Pesticide Use by Cotton Farmers in China. J. Dev. Econ..

[13] Zhou W., Li M., Achal V. (2024). A Comprehensive Review on Environmental and Human Health Impacts of Chemical Pesticide Usage. Emerg. Contam..

[14] Sun S., Li C., Chee P.W., Paterson A.H., Meng C., Zhang J., Ma P., Robertson J.S., Adhikari J. (2021). High Resolution 3D Terrestrial LiDAR for Cotton Plant Main Stalk and Node Detection. Comput. Electron. Agric..

[15] Manavalan R. (2022). Towards an Intelligent Approaches for Cotton Diseases Detection: A Review. Comput. Electron. Agric..

[16] Wang Z., Wang R., Wang M., Lai T., Zhang M. (2024). Self-Supervised Transformer-Based Pre-Training Method with General Plant Infection Dataset. Proceedings of the Chinese Conference on Pattern Recognition and Computer Vision (PRCV).

[17] Rothe P.R., Kshirsagar R.V. Automated Extraction of Digital Images Features of Three Kinds of Cotton Leaf Diseases. Proceedings of the 2014 International Conference on Electronics, Communication and Computational Engineering (ICECCE).

[18] Jenifa A., Ramalakshmi R., Ramachandran V. Classification of Cotton Leaf Disease Using Multi-Support Vector Machine. Proceedings of the 2019 IEEE International Conference on Intelligent Techniques in Control, Optimization and Signal Processing (INCOS).

[19] Kurale N.G., Vaidya M.V. Classification of Leaf Disease Using Texture Feature and Neural Network Classifier. Proceedings of the 2018 International Conference on Inventive Research in Computing Applications (ICIRCA).

[20] Ebrahimi M., Khoshtaghaza M.H., Minaei S., Jamshidi B. (2017). Vision-Based Pest Detection Based on SVM Classification Method. Comput. Electron. Agric..

[21] Fu H., Zhao H., Song R., Yang Y., Li Z., Zhang S. (2022). Cotton Aphid Infestation Monitoring Using Sentinel-2 MSI Imagery Coupled with Derivative of Ratio Spectroscopy and Random Forest Algorithm. Front. Plant Sci..

[22] Wang R.-F., Su W.-H. (2024). The Application of Deep Learning in the Whole Potato Production Chain: A Comprehensive Review. Agriculture.

[23] Yang Z.-Y., Xia W.-K., Chu H.-Q., Su W.-H., Wang R.-F., Wang H. (2025). A Comprehensive Review of Deep Learning Applications in Cotton Industry: From Field Monitoring to Smart Processing. Plants.

[24] Zhou G., Wang R.-F. (2025). The Heterogeneous Network Community Detection Model Based on Self-Attention. Symmetry.

[25] Cui K., Zhu R., Wang M., Tang W., Larsen G.D., Pauca V.P., Alqahtani S., Yang F., Segurado D., Lutz D. (2025). Detection and Geographic Localization of Natural Objects in the Wild: A Case Study on Palms. arXiv.

[26] Pan C.-H., Qu Y., Yao Y., Wang M.-J.-S. (2024). HybridGNN: A Self-Supervised Graph Neural Network for Efficient Maximum Matching in Bipartite Graphs. Symmetry.

[27] Zhao C.-T., Wang R.-F., Tu Y.-H., Pang X.-X., Su W.-H. (2024). Automatic Lettuce Weed Detection and Classification Based on Optimized Convolutional Neural Networks for Robotic Weed Control. Agronomy.

[28] Hu P., Cai C., Yi H., Zhao J., Feng Y., Wang Q. (2022). Aiding Airway Obstruction Diagnosis with Computational Fluid Dynamics and Convolutional Neural Network: A New Perspective and Numerical Case Study. J. Fluids Eng..

[29] Cui K., Tang W., Zhu R., Wang M., Larsen G.D., Pauca V.P., Alqahtani S., Yang F., Segurado D., Fine P. (2024). Real-Time Localization and Bimodal Point Pattern Analysis of Palms Using Uav Imagery. arXiv.

[30] Wu A.-Q., Li K.-L., Song Z.-Y., Lou X., Hu P., Yang W., Wang R.-F. (2025). Deep Learning for Sustainable Aquaculture: Opportunities and Challenges. Sustainability.

[31] Qiu K., Zhang Y., Ren Z., Li M., Wang Q., Feng Y., Chen F. (2024). SpemNet: A Cotton Disease and Pest Identification Method Based on Efficient Multi-Scale Attention and Stacking Patch Embedding. Insects.

[32] Zhang T., Zhu J., Zhang F., Zhao S., Liu W., He R., Dong H., Hong Q., Tan C., Li P. (2024). Residual Swin Transformer for Classifying the Types of Cotton Pests in Complex Background. Front. Plant Sci..

[33] Faisal H.M., Aqib M., Rehman S.U., Mahmood K., Obregon S.A., Iglesias R.C., Ashraf I. (2025). Detection of Cotton Crops Diseases Using Customized Deep Learning Model. Sci. Rep..

[34] Feng H., Chen X., Duan Z. (2025). LCDDN-YOLO: Lightweight Cotton Disease Detection in Natural Environment, Based on Improved YOLOv8. Agriculture.

[35] Zhang Y., Ma B., Hu Y., Li C., Li Y. (2022). Accurate Cotton Diseases and Pests Detection in Complex Background Based on an Improved YOLOX Model. Comput. Electron. Agric..

[36] Kakade K.J., More V.A., Shinde M., Suryawanshi K., Shinde G.U. Design of Precision Agriculture System Using Automating Pink Bollworm Detection in Cotton Crops: AI Based Digital Approach for Sustainable Pest Management. Proceedings of the 2025 1st International Conference on AIML-Applications for Engineering & Technology (ICAET).

[37] Mehta S., Rastegari M. (2021). Mobilevit: Light-Weight, General-Purpose, and Mobile-Friendly Vision Transformer. arXiv.

[38] Mehta S., Rastegari M. (2022). Separable Self-Attention for Mobile Vision Transformers. arXiv.

[39] Wadekar S.N., Chaurasia A. (2022). Mobilevitv3: Mobile-Friendly Vision Transformer with Simple and Effective Fusion of Local, Global and Input Features. arXiv.

[40] He K., Zhang X., Ren S., Sun J. Deep Residual Learning for Image Recognition. Proceedings of the 2016 IEEE Conference on Computer Vision and Pattern Recognition (CVPR).

[41] Simonyan K., Zisserman A. (2014). Very Deep Convolutional Networks for Large-Scale Image Recognition. arXiv.

[42] Tan M., Le Q. Efficientnet: Rethinking Model Scaling for Convolutional Neural Networks. Proceedings of the International Conference on Machine Learning, PMLR.

[43] Howard A., Sandler M., Chen B., Wang W., Chen L.-C., Tan M., Chu G., Vasudevan V., Zhu Y., Pang R. Searching for MobileNetV3. Proceedings of the 2019 IEEE/CVF International Conference on Computer Vision (ICCV).

[44] Xin J., Tang R., Lee J., Yu Y., Lin J. (2020). DeeBERT: Dynamic Early Exiting for Accelerating BERT Inference. arXiv.

[45] Korol G., Beck A.C.S. (2025). IoT–Edge Splitting with Pruned Early-Exit CNNs for Adaptive Inference. IEEE Trans. Very Large Scale Integr. (VLSI) Syst..

[46] Gilbert M.S., Pacheco R.G., Couto R.S., Fladenmuller A., Dias de Amorim M., de Campos M.L.R., Campista M.E.M. Early-Exit Criteria for Edge Semantic Segmentation. Proceedings of the 2025 IEEE International Conference on Machine Learning for Communications and Networking.

[47] Jacob B., Kligys S., Chen B., Zhu M., Tang M., Howard A., Adam H., Kalenichenko D. Quantization and Training of Neural Networks for Efficient Integer-Arithmetic-Only Inference. Proceedings of the 2018 IEEE/CVF Conference on Computer Vision and Pattern Recognition.

[48] Xiao G., Lin J., Seznec M., Wu H., Demouth J., Han S. SmoothQuant: Accurate and Efficient Post-Training Quantization for Large Language Models. Proceedings of the 40th International Conference on Machine Learning, PMLR.

[49] Shi H., Cheng X., Mao W., Wang Z. (2024). P2-ViT: Power-of-Two Post-Training Quantization and Acceleration for Fully Quantized Vision Transformer. IEEE Trans. Very Large Scale Integr. (VLSI) Syst..

[50] Touvron H., Cord M., Douze M., Massa F., Sablayrolles A., Jegou H. Training Data-Efficient Image Transformers & Distillation through Attention. Proceedings of the 38th International Conference on Machine Learning, PMLR.

[51] Pan J., Bulat A., Tan F., Zhu X., Dudziak L., Li H., Tzimiropoulos G., Martinez B., Avidan S., Brostow G., Cissé M., Farinella G.M., Hassner T. (2022). EdgeViTs: Competing Light-Weight CNNs on Mobile Devices with Vision Transformers. Proceedings of the Computer Vision—ECCV 2022.

[52] Wang R.-F., Tu Y.-H., Chen Z.-Q., Zhao C.-T., Su W.-H. A Lettpoint-Yolov11l Based Intelligent Robot for Precision Intra-Row Weeds Control in Lettuce. https://papers.ssrn.com/sol3/papers.cfm?abstract_id=5162748.

[53] Bishshash P., Nirob A.S., Shikder H., Sarower A.H., Bhuiyan T., Noori S.R.H. (2024). A Comprehensive Cotton Leaf Disease Dataset for Enhanced Detection and Classification. Data Brief.

[54] Paul Joshua K., Alex S.A., Mageswari M., Jothilakshmi R. (2025). Enhanced Conditional Self-Attention Generative Adversarial Network for Detecting Cotton Plant Disease in IoT-Enabled Crop Management. Wirel. Netw..

[55] Yang Z.-X., Li Y., Wang R.-F., Hu P., Su W.-H. (2025). Deep Learning in Multimodal Fusion for Sustainable Plant Care: A Comprehensive Review. Sustainability.

